# Incorporation of Functionalized Halloysite Nanotubes (HNTs) into Thin-Film Nanocomposite (TFN) Nanofiltration Membranes for Water Softening

**DOI:** 10.3390/membranes13020245

**Published:** 2023-02-18

**Authors:** Amirsajad Atashgar, Daryoush Emadzadeh, Somaye Akbari, Boguslaw Kruczek

**Affiliations:** 1 Department of Chemical and Biological Engineering, University of Ottawa, Ottawa, ON K1N 6N5, Canada; 2 Textile Engineering Department, Amirkabir University of Technology, 424 Hafez Ave., Tehran P.O. Box 15875-4413, Iran

**Keywords:** thin-film nanocomposite membranes, nanofiltration, modified halloysite nanotubes, first generation of poly(amidoamine) dendrimers

## Abstract

Incorporating nanoparticles (NPs) into the selective layer of thin-film composite (TFC) membranes is a common approach to improve the performance of the resulting thin-film nanocomposite (TFN) membranes. The main challenge in this approach is the leaching out of NPs during membrane operation. Halloysite nanotubes (HNTs) modified with the first generation of poly(amidoamine) (PAMAM) dendrimers (G1) have shown excellent stability in the PA layer of TFN reverse-osmosis (RO) membranes. This study explores, for the first time, using these NPs to improve the properties of TFN nanofiltration (NF) membranes. Membrane performance was evaluated in a cross-flow nanofiltration (NF) system using 3000 ppm aqueous solutions of MgCl_2_, Na_2_SO_4_ and NaCl, respectively, as feed at 10 bar and ambient temperature. All membranes showed high rejection of Na_2_SO_4_ (around 97–98%) and low NaCl rejection, with the corresponding water fluxes greater than 100 L m^−2^ h^−1^. The rejection of MgCl_2_ (ranging from 82 to 90%) was less than that for Na_2_SO_4_. However, our values are much greater than those reported in the literature for other TFN membranes. The remarkable rejection of MgCl_2_ is attributed to positively charged HNT-G1 nanoparticles incorporated in the selective polyamide (PA) layer of the TFN membranes.

## 1. Introduction

Membrane separation is widely used for water desalination [1], solvent purification [2], and wastewater treatment in different industries such as petrochemical [3], food [4], etc. Pressure-driven processes such as microfiltration (MF), ultrafiltration (UF) [5,6], nanofiltration (NF) [7,8], and reverse osmosis (RO) [9] are commonly used technologies. The RO membranes, which can reject monovalent salts, are the most selective membranes for pressure-driven processes. Still, they require high operating pressures and hence are energy-intensive. Although not suitable for the rejection of monovalent salts, NF membranes can effectively separate multivalent salts and heavy metals from aqueous solutions and require lower operating pressures. At the same time, NF membranes are more permeable than their RO counterparts. Therefore, NF, which is also referred to as low-pressure RO, has become an exciting field of research in membrane separation technology used for water desalination, solvent purification, and wastewater treatment [9,10,11].

The pore size of NF membranes is between UF membranes and RO membranes. Generally, the NF membranes have a negatively charged surface; therefore, in addition to the size exclusion, they can reject solutes, such as different inorganic salts and organic molecules, based on electrostatic repulsion (Donnan effect) [12,13]. In the Donnan effect, the negatively charged surface membranes attract cations and repulse anions. The higher the valence, the stronger the attraction or rejection of the ions by the membrane. At the same time, the feed and permeate maintain electroneutrality. In other words, although the negatively charged NF membranes attract cations, their transport across the membrane is limited because of the rejection of their corresponding anions. The separation properties of NF membranes can be tailored by adjusting their surface charge depending on the solute to be rejected. 

NF membranes can be categorized into two groups: integrally skinned asymmetric and thin-film composite (TFC) structures. The first group is mainly formed with a phase-inversion process, while the standard method for preparing TFC NF membranes is interfacial polymerization (IP) [14]. TFC membranes, consisting of an ultra-thin selective polyamide (PA) layer and porous polymeric support, are widely used as NF membranes and dominate the market. To optimize TFC membrane performance, the porous support structure and the selective PA layer can be independently modified [15,16]. The performance of TFC membranes can be improved by incorporating nanoparticles (NPs) such as halloysite nanotubes (HNTs), titanium oxide (TiO_2_), titanium nanotubes (TNTs), and carbon nanotubes (CNTs) into the support or the selective layer of the membrane. The resulting membranes represent a separate category of membranes referred to as thin-film nanocomposite (TFN) membranes [17,18,19,20,21,22].

Despite great potential, there are also some significant problems with TFN membranes; for example, the nanoparticles (NPs) tend to aggregate, resulting in uneven distribution of the NPs in the selective layer and leading to defects in the membrane structure [22]. Another critical challenge for TFN membranes is that the NPs could have a toxic effect on the environment and living organisms if they leach out of the membrane. One method for overcoming all of the above-stated problems is the surface modification of the NPs. By functionalizing the NPs, they can be better dispersed in the selective layer, resulting in more stable and selective TFN membranes [20]. 

HNTs with a molecular formula of Al_2_Si_2_O_5_(OH)4·nH_2_O have natural multi-layer aluminosilicates. They are an excellent nanofiller candidate for polymeric membranes for water treatment applications due to their low production cost, unique structure, and minimal environmental risk [23]. HNTs’ exterior surface is composed of siloxane groups with some hydroxyl groups, which enables their functionalization [22,24]. In turn, the functionalization of HNTs allows modification of the surface charge of the resulting TFN membrane. In some studies, HNTs were used to synthesize TFN forward osmosis (FO) and NF membranes, which improved the membranes’ antifouling properties and performance [25,26]. For example, Ghanbari et al. used HNTs for water desalination with TFN FO membranes [20]. They reported that by incorporating 0.05% (*w*/*v*) of HNTs into the selective layer, water flux increased by 50%. Ormanci-Acar et al. fabricated TFN NF membranes by incorporating different loadings of HNTs in the membranes’ selective layer [25]. They evaluated the rejection of their TFN NF membranes with MgSO_4_ and observed that the resultant TFN membrane showed increased water flux without a noticeable salt rejection loss.

Poly(amidoamine) (PAMAM) dendrimers have a hyperbranched hydrophilic structure consisting of many amine groups. PAMAM dendrimers have high hydrophilicity. Grafting them directly or through NPs into a membrane can increase membrane hydrophilicity and enhance antifouling properties [27,28]. Asempour et al. fabricated RO TFN membranes by incorporating modified HNTs with the first generation of poly(amidoamine) (PAMAM) dendrimers into the selective layer. They reported that the resulting membranes were more stable than TFN membranes with unmodified HNTs. Furthermore, they observed higher water flux without sacrificing salt rejection in brackish water desalination compared to other TFN and reference TFC membranes [22].

In this study, we hypothesized that the approach used by Asempour et al. to improve the performance and stability of TFN RO membranes is also applicable to TFN NF membranes. We synthesized TFC and TFN membranes by in situ interfacial polymerization (IP) of piperazine (PIP) and 1,3,5-benzenetricarbonyl trichloride (TMC) on commercial ultrafiltration (UF) membranes (PS35). We dispersed different loadings of modified HNTs with PAMAM dendrimers into the TMC solution before the IP when preparing the TFN membranes. The modified NPs and NF membranes were thoroughly characterized using different methods, including membrane performance tests. 

## 2. Materials and Methods

### 2.1. Materials

Solecta, Inc. (Oceanside, CA, USA) donated a polysulfone ultrafiltration membrane (PS35) with a molecular weight cutoff of 20,000 Da. Piperazine (PIP), 1,3,5-benzenetricarbonyl trichloride (TMC), n-hexane, aminopropyltriethoxysilane (APTES), ethanol, diethyl ether, dimethylformamide (DMF), ethylenediamine (EDA), magnesium chloride (MgCl_2_), sodium sulphate (Na_2_SO_4_), and sodium chloride (NaCl) were all laboratory grade and were purchased from Sigma-Aldrich (Saint Louis, MO, USA). Delta (Dolsk, Poland) provided HNTs. Deionized water was used to prepare the PIP solution, and distilled water was used to wash the membranes and prepare aqueous feed solutions.

### 2.2. Functionalization of HNTs

**Figure 1:**
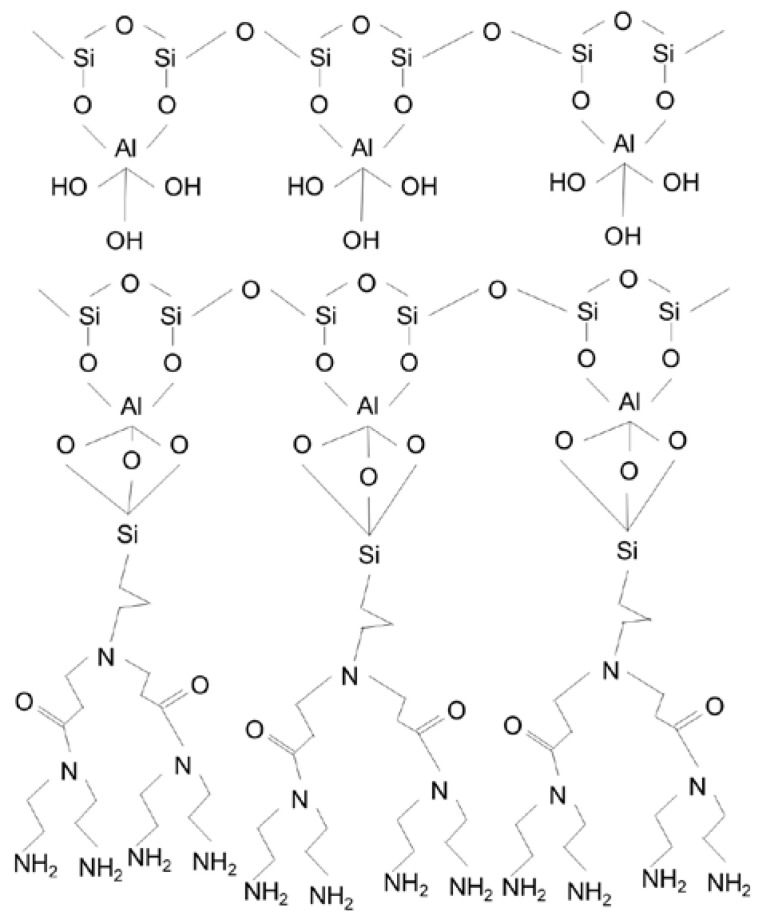
Schematic structure of the HNTs-G1 [22].

The detailed synthesis procedure for dendrimers and functionalization of HNT are fully described elsewhere [29]. Briefly, the first step of the functionalization was acid treatment to remove all contaminants. In this step, a mixture of HNTs and aqueous HCl solution (35%) were magnetically stirred for 24 h and then washed with distilled water. For the HNTs’ amine functionalization (HNT-NH_2_), dry-acid-treated HNTs were refluxed with an APTES solution in toluene (4/15 (*v*/*v*)) for 12 h at 60 °C. To synthesize the first generation of the PAMAM dendrimers on the HNTs (HNTs-G1), 32.15 g of the HNTs-NH_2_ was reacted (Michael reaction) with methyl acrylate (15 mL) in an ethanol solution at 60 °C for 24 h. The final solution was put into a centrifuge to separate the HNTs. Before drying at 60 °C, the HNTs were washed and centrifuged with diethyl ether, ethanol, and methanol to remove impurities. In the next step, ethylene diamine was added to the mixture of HNT in ethanol and stirred at 60 °C for 24 h. After the separation of the HNT by centrifugation, the resulting NPs, HNT-G1, were washed with distilled water and dried. Figure 1 shows the chemical structure of the functionalized HNTs.

### 2.3. Synthesis of TFC and TFN Membranes

A PS35 UF membrane was used as a substrate for the thin PA layer formation, synthesized by in situ interfacial polymerization of the PIP and TMC monomers. Fabrication of the TFC membrane started with pouring a 2% (*w*/*v*) solution of PIP in deionized water onto the substrate. After 5 min, the excess solution was drained off using a Teflon roller (any visible droplets were removed from the surface). This was followed by pouring 0.05% (*w*/*v*) of TMC in n-hexane onto the support for 1 min; the excess solution was drained off, and the membrane was rinsed thoroughly with n-hexane. The membranes were then placed in an oven for 10 min at 95 °C. Finally, the TFC membranes were rinsed with distilled water and stored in deionized water. The only difference between synthesizing the TFC and HNT-based TFN membranes is their TMC solution: different amounts of NPs were dispersed in the TMC solution in the n-hexane solution (Table 1). To minimize NP agglomeration, the resulting suspension was sonicated for 1 h before IP. The TFN membranes were named based on the concentration of HNTs-G1 in the solution of TMC in n-hexane.

### 2.4. Nanofiltration Performance

To evaluate the solute rejection and water flux of the membranes, they were tested in a cross-flow system with three parallel membrane cells. Each cell had an effective area for permeation of 17.54 cm^2^. The system is described elsewhere [30]. The feed solution’s temperature and pressure were 25 ± 2 °C and 10–20 ± 0.1 bar, respectively. The feed flow rate was controlled at 2.4 ± 0.2 L/min. Permeate flux (*J*) [L m^−2^ h^−1^] was calculated using Equatiom (1), using the volume of collected permeate (*V*) [L] per area of the membrane (*A*) [m^2^] and time (*t*) [h]. Membrane selectivity was evaluated using 3000 ppm single-solute aqueous solutions, including MgCl_2_, Na_2_SO_4_, and NaCl. Salt rejection (*R*) was calculated using Equation (2), in which *C_p_* and *C_f_* are the salt concentrations of permeate and feed, respectively. The concentrations of feed and permeate solution were measured using an Oakton CON 6+ conductivity meter. Table 2 lists the hydrated radii salt ions used in membrane performance tests. The pH of the salt solutions was in the range of 6–7.

(1) $$J=\frac{V}{A\times t}$$

(2) $$R=\frac{C_{f}-C_{p}}{C_{f}}\times 100\%$$

### 2.5. Membrane and Nanoparticle Physical Characterization

To determine the percentage of functionalization of the HNTs, a Q5000 thermal gravimetric analyzer (TGA) (TA Instruments Ltd., New Castle, DE, USA) was used. In a nitrogen atmosphere, the heating rate was 10 °C/min from 30 °C to 800 °C. The top surface morphology of the membranes was evaluated using scanning electron microscopy (SEM). All of the samples were coated by gold sputtering. Attenuated Total Reflection-Fourier Transform Infrared (ATR-FTIR) spectra of the membranes (TFC and TFN membranes) and NPs were obtained using a Nicolet 6700 FT-IR instrument with a diamond crystal (Thermo Fisher, Waltham, MA, USA). OMNIC ™ software was utilized to analyze the spectra. To investigate the TFC and TFN membranes’ hydrophilicity, a VCA Optima goniometer (AST Products, Inc., Billerica, MA, USA) was used. The morphology of the nanoparticle was characterized using a TEM (Philips CM30, Algonquin, IL, USA) instrument. The zeta potential of the surface of membranes was determined using a zeta analyzer (Zetasizer PSS0012-22, Malvern Instruments, Westborough, MA, USA).

## 3. Results and Discussion

### 3.1. Characterization of the Nanoparticles

**Figure 2:**
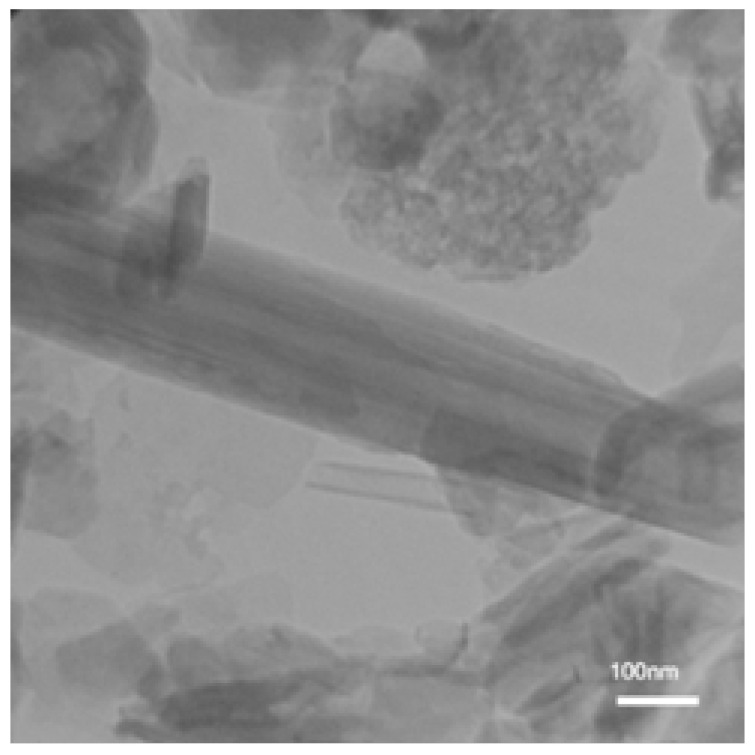
TEM image of acid-washed HNTs [22].

**Figure 3:**
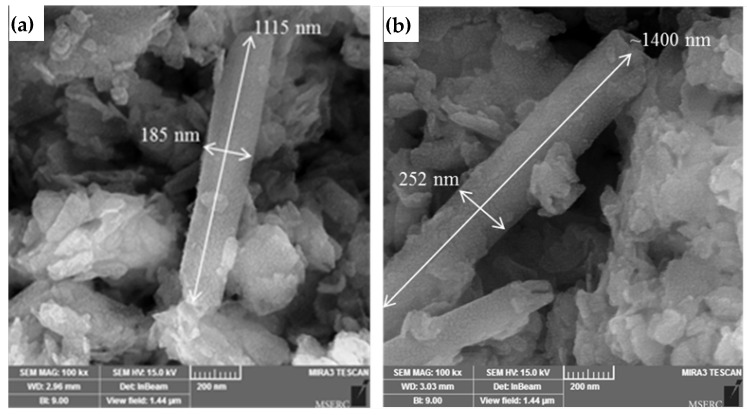
SEM images of (**a**) HNTs (**b**) HNTs-G1 [22].

The characterization of NPs is similar to our previous work [22]. Figure 2 shows the TEM image of the acid-washed HNTs. It is known that HNTs are heterogeneous in size [32]. HNTs have a tubular structure with inner and outer diameters of 28 nm and 170 nm, respectively. Figure 3 presents SEM images of the acid-washed HNTs and HNTs-G1. The arrows in Figure 3 show the diameter and length of the HNTs. The modification of the HNTs appears not to change their morphology or structure.

The effects of the modification on the surface charge of the NPs were investigated using zeta potential. The summary of the zeta potential analysis is presented in Table 3. Due to the existence of the hydroxyl groups, the surface of the unmodified HNTs is negatively charged. After functionalization with the first generation of PAMAM dendrimers, the zeta potential of HNTs-G1 increases to +2.2 mV. The changes in zeta potential after modification of the HNTs indicate the presence of amino-functionalized groups on the surface of the HNTs in the first generation of PAMAM dendrimers.

A summary of the TGA analysis is also presented in Table 3. The percentage of weight loss at 800 °C of HNTs-G1 (22.94%) is greater than that of the HNTs (17.65%). Subtracting the percentage of weight loss of HNTs from the percentage of weight loss of HNTs-G1 yields 5.29%, a measure of the organic weight increment resulting from the functionalization of HNTs with the first generation of PAMAM dendrimers.

**Figure 4:**
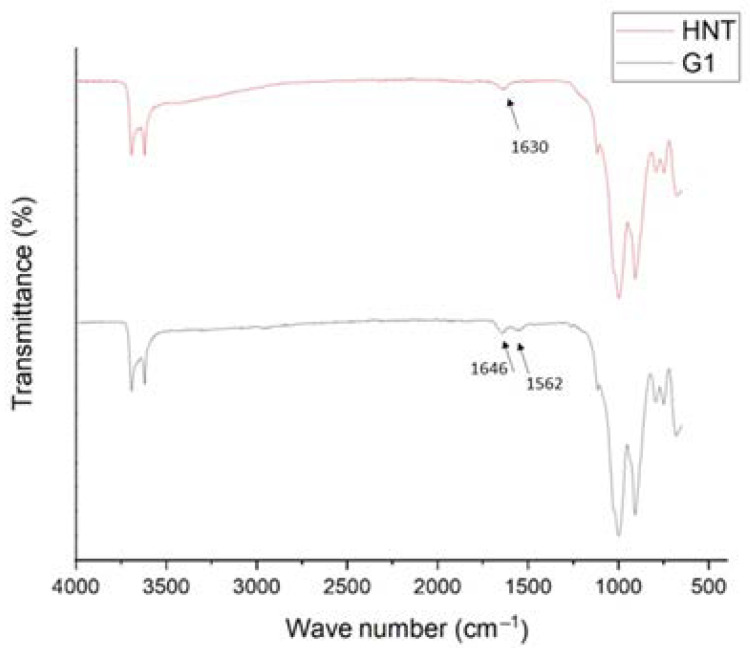
ATR-FTIR spectra of HNTs and HNTs-G1. The arrow shows the presence of a new peak according to the amide group.

ATR-FTIR analysis is an appropriate way to evaluate the chemical reaction that occurs when modifying the HNTs. Figure 4 shows the ATR-FTIR spectra of the HNTs and HNTs-G1. The peaks of the HNTs, which appear at 3621 and 3695 cm^−1^, are the stretching vibration of the inner surface of Al-OH groups [22]. The peaks at 1630 and 910 cm^−1^ are ascribed to the OH bending vibrations associated with the interlayer molecules of HNTs of water and Al-OH, respectively. The peak related to the stretching bond of Si-O appears at 1030 cm^−1^. In the spectrum of the HNTs-G1, two new peaks appear at 1646 and 1562 cm^−1^. The peak at 1646 cm^−1^ is associated with the C=O valence vibration, and the peak at 1562 cm^−1^ is associated with the N-H bending vibration. Therefore, Figure 4 confirms the expected chemical reaction associated with the functionalization of HNTs with the first generation of PAMAM dendrimers.

From the results presented in this section, it is evident that the functionalization of HNTs with the first generation of PAMAM dendrimers was successful.

### 3.2. Characterization of the Membranes

**Figure 5:**
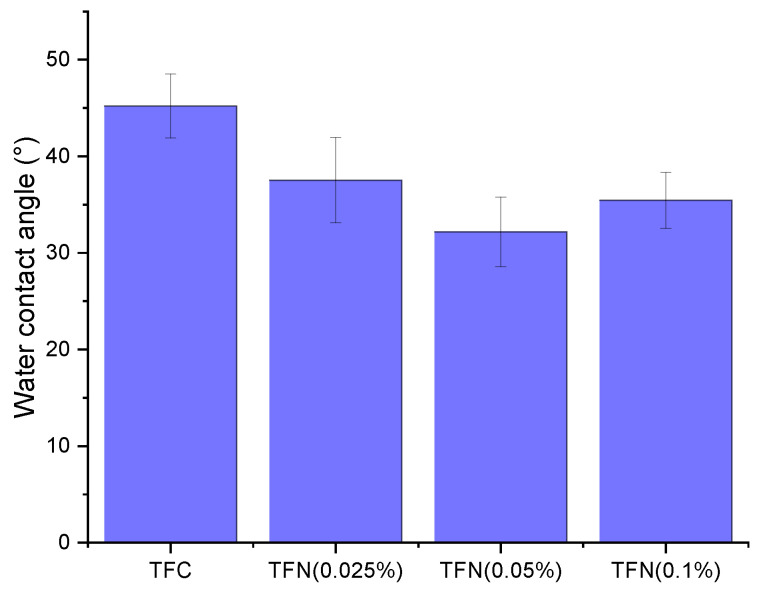
Top surface water contact angle of TFC and TFN membranes.

The results of the contact angle measurement are illustrated in Figure 5. The reported values and their corresponding error bars represent the average and standard deviation for at least 10 droplets of DI water, each with a volume of 2 µL, for a given type of membrane. Despite some overlapping of the error bars, it is evident that the incorporation of HNTs-G1 has decreased the water contact angle, indicating an increase in the surface hydrophilicity of the resulting membranes. The TFN membranes’ lowest and highest water contact angles are for the TFN (0.05%) and the TFN (0.025%), respectively. The increase in contact angle for the 0.1% membrane could be related to the agglomeration of HNTs-G1. Agglomeration of NPs could result in more exposure of silane groups, leading to a decrease in the hydrophilicity of agglomerated HNTs.

**Figure 6:**
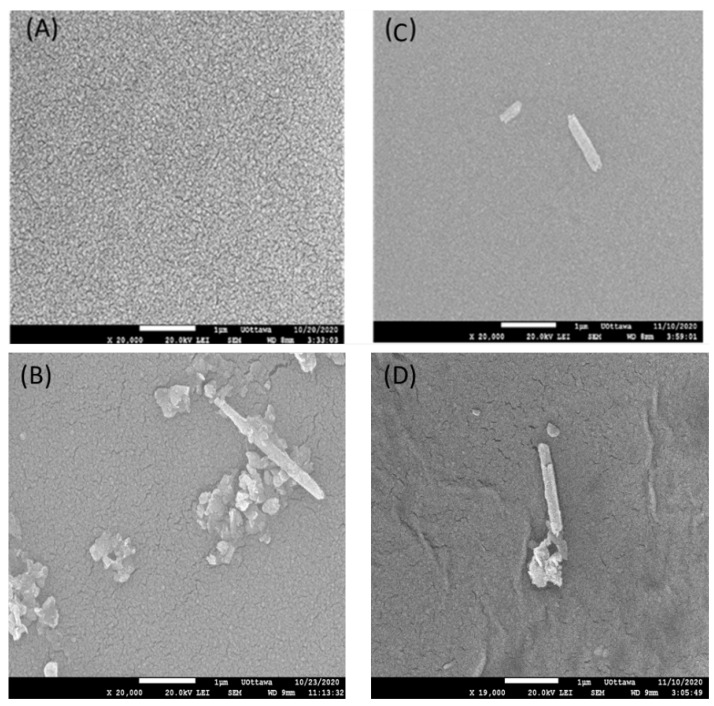
SEM images of the top surface of (**A**) TFC, (**B**) TFN (0.025%), (**C**) TFN (0.05%), and (**D**) TFN (0.1%) membranes.

Figure 6 shows SEM images of the top surface TFC and TFN membranes. The surface of the TFC membrane appears to have a uniform nodular structure. Incorporating HNTs-G1 leads to the appearance of horizontal cylindrical particles on the top surface, which could be the NPs. There are also some irregular particles on the surface of the TNF-G1 membranes (Figure 6B,D), which could be agglomerates of HNTs penetrating the top surface. Interestingly, these distinctive features are most evident on the TFN-G1 membrane with the lowest percentage of NPs. While the TFN membrane’s surface retains a nodular structure in the background, the size of the nodules is smaller than that on the TFC membrane surface. Moreover, as the percentage of HNTs-G1 increases, the membrane surface becomes less uniform. The nodular structure is typical for membranes that are synthesized by interfacial polymerization [33,34].

**Figure 7:**
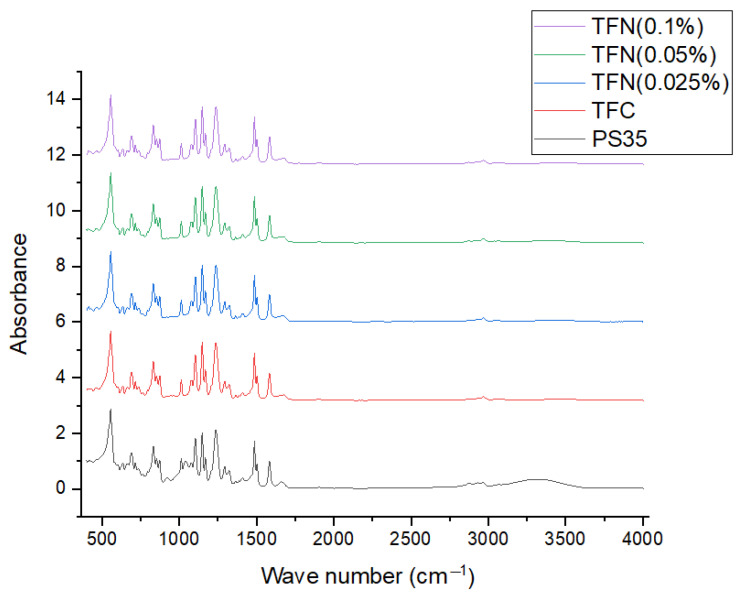
FTIR spectra of the PS35 (support), TFC, and TFN membranes.

Figure 7 presents the FTIR spectra for the PS35, TFC, and TFN membranes. To normalize the membranes’ FTIR spectra, each spectrum’s peak was divided by the intensity of the internal reference, e.g., the peak at 1584 cm^−1^, which is attributed to the polysulfone. Table 4 presents normalized intensities for all ranges corresponding to amide Ι (1618–1720 cm^−1^), aromatic amide (1600–1620 cm^−1^), and various peaks in the 3120–3706 cm^−1^ range corresponding to the PS35 support. Interestingly, the amide peaks are also present in the PS35 support. This is probably due to the manufacturer’s modification of the polysulfone to enhance the substrate’s water flux [22]. Moreover, the intensity of amide peaks on the PS35 support is lower than on the TFC and TFN membranes. Therefore, based on the results in Table 4, it can be concluded that the PA layer was formed in all TFC and TFN membranes.

**Figure 8:**
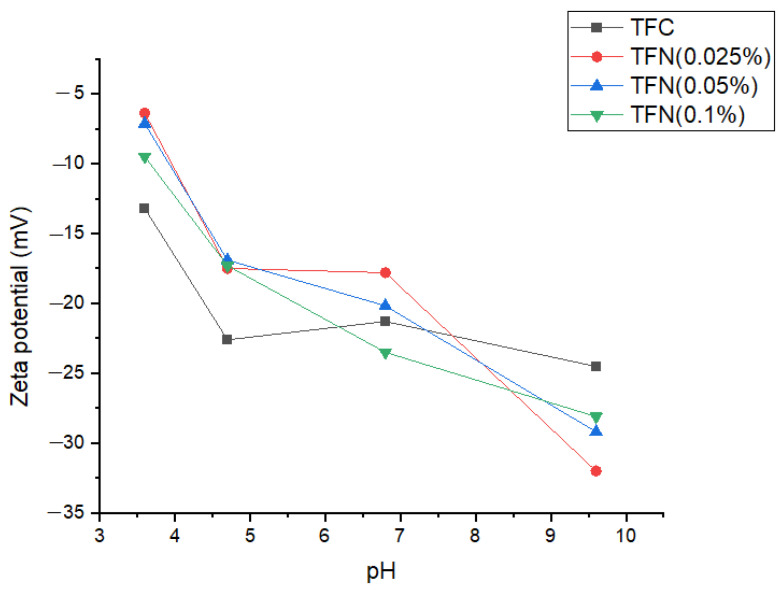
Surface zeta potential of TFC and TFN membranes.

The surface charge of the TFC and TFN membranes was evaluated by measuring their surface zeta potential at different pH levels. In turn, the zeta potential of the selective layer influences the solute rejection by the NF membrane. Figure 8 summarizes the zeta potential results. In general, the zeta potential of all membranes decreases with an increase in pH. In other words, the surface charge in a basic environment is greater than in an acidic one. At acidic pH levels, the TFN membranes’ zeta potential is less negative than that of the TFC membranes. There is no clear trend at neutral pH. However, at basic pH levels, the surface charge of the TFN membranes is greater than that of the TFC membranes. This phenomenon can be attributed to the amine groups incorporating HNTs functionalized with the first generation of PAMAM dendrimers. The zeta potential results agree with the enhanced membrane hydrophilicity (Figure 5) [35]. The residual COCl functional groups in the TMC monomer are turned into carboxylic acid groups (COOH) by hydrolyzation in the aqueous solution, which results in negatively charged membranes [36].

### 3.3. NF Performance of the Membranes

**Figure 9:**
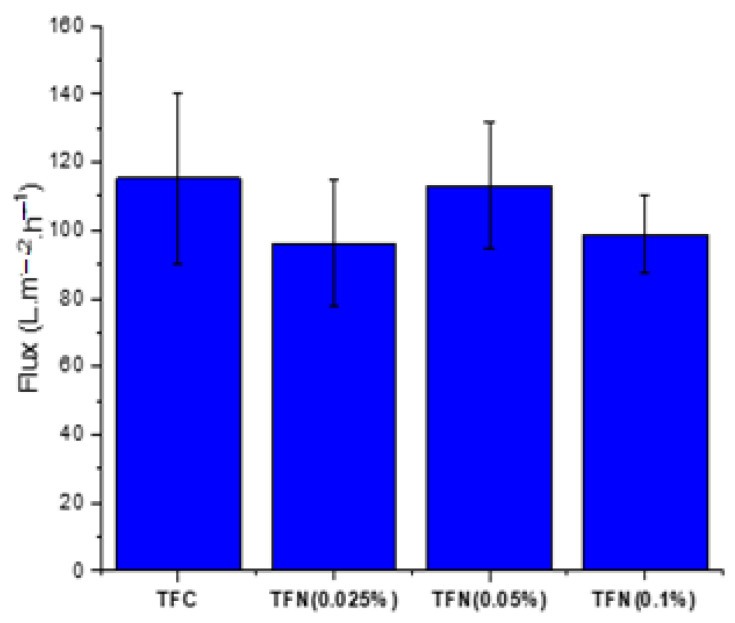
Pure water flux performance of TFC and TFN membranes at 20 bar and 25 °C.

The TFC and TFN NF membranes’ separation performance was examined with a 3000 ppm feed solution of different salts at 10 bar and 25 °C. Figure 9 shows the pure water flux of the TFC and TFN membranes. The reported values and the corresponding error bars represent the average and standard deviation for at least 4 coupons for a given type of membrane. The water flux of the pristine TFC membrane of 115 L m^−2^ h^−1^ is higher than that for the TFN membranes. The lowest water flux of 96 L m^−2^ h^−1^ is observed for the TFN membrane with the lowest loading of NPs (0.025%). The contact angle measurements (Figure 5) show that the TFN membranes are more hydrophilic than the reference TFC membrane. Therefore, the expected trend of increased water flux with increased surface hydrophilicity of the membrane is not observed. It is important to note that in addition to surface hydrophilicity, water flux is also affected by a membrane’s pore size and surface roughness. On the other hand, considering only the TFN membranes, the water flux correlates very well with these membranes’ water contact angle. It is important to note that considering the magnitude of the error bars, the differences between different membranes’ water fluxes are insignificant.

**Figure 10:**
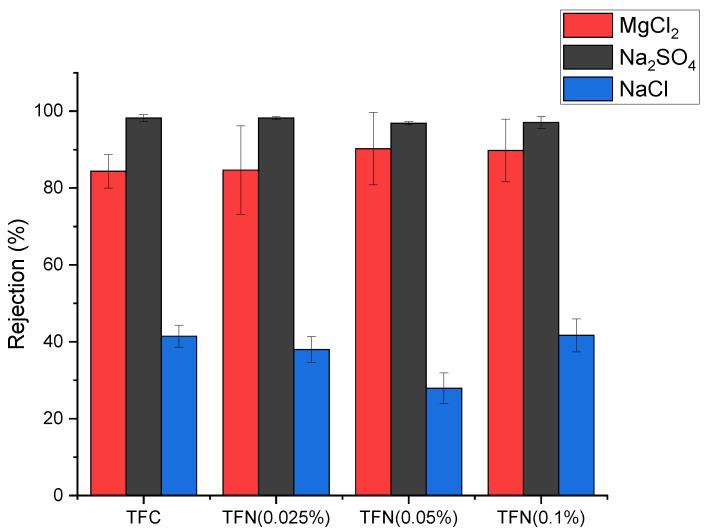
Rejection of different salts by TFC and TFN using 3000 ppm feed aqueous solution at 10 bar and 25 °C.

Figure 10 shows the TFC and TFN membranes’ rejection of inorganic salts (MgCl_2_, Na_2_SO_4_, NaCl). As expected, the rejection of NaCl by all membranes is very low (around or below 40%). NF membranes are not suitable for the separation of monovalent salts. The general trend for rejection is Na_2_SO_4_ > MgCl_2_ > NaCl. Although NaCl and Na_2_SO_4_ have the same monovalent cation (Na^+^), the hydration radius of SO_4_^2−^ is higher than that of Cl^−^ [33,34]. However, the difference in the hydration radii, i.e., size exclusion, is not the main reason for the difference in the rejection of NaCl and Na_2_SO_4_. As seen in Figure 8, all membranes fabricated in this study were negatively charged, which is generally the case for NF membranes. The negatively charged NF membranes have a higher rejection of divalent SO_4_^2−^ anions than of monovalent Cl^−^ anions due to stronger electrostatic repulsion. Electroneutrality of the feed solution thus requires corresponding rejection of the Na^+^ cations. Therefore, the Donnan effect is the main reason for the vast difference between the rejection of NaCl and Na_2_SO_4_. The size exclusion also contributes to the rejection of dissolved salts in the NF process. For example, MgCl_2_ and NaCl have the same anion (Cl^−^), but the divalent Mg^2+^ has a larger hydration radius than the Na^+^; therefore, MgCl_2_ rejection is higher than NaCl because of size exclusion [25,31,36].

The dominance of the Donnan effect over size exclusion in NF filtration explains the higher rejection of Na_2_SO_4_ than MgCl_2_. As seen in Table 2, Mg^2+^ has the highest hydration radius among all ions. However, since the membranes are negatively charged, the rejection of the salts must be primarily determined by the rejection of the anions. In contrast, the rejection of the corresponding cations must follow accordingly to maintain the feed solution’s electroneutrality.

It is important to note that the rejection of Na_2_SO_4_ by TFC and TFN membranes is similar. As shown in Figure 8, there is no clear trend between nanoparticle loading and surface zeta potential, and the surface charge is negative. Consequently, the repulsion of SO_4_^2−^ ions, the main contributor to the rejection of Na_2_SO_4_ by these membranes, is similar.

Table 5 compares the performance of our TFN membranes based on HNTs-G1 with TFN membranes based on other nanomaterials. Because NF tests can be carried out at different feed pressures, the productivity of the membranes in Table 5 is compared based on water permeance rather than water flux. Water permeance is a pressure-gradient-normalized water flux. It can be noticed that many membranes reported in Table 5 are more productive than our TFN membranes, and some also exhibit comparable or even higher rejection of Na_2_SO_4_. However, compared to other membranes listed in Table 5, our TFN membranes exhibit very high rejection of MgCl_2_. Only the poly(dopamine) MWCNT-based TFN membranes reported by Zhao et al. [37] exhibit similar rejection to our membranes. These authors claim that high rejection of MgCl_2_ by poly(dopamine) MWCNT-based TFN membranes is due to the positive surface charge of the membrane. Consequently, the Donnan effect arises from the repulsion of divalent Mg^2+^ cations rather than monovalent Cl^-^ anions. As shown in Figure 8, our TFC and TFN membranes are negatively charged. However, since the HNTs-G1 are positively charged (Table 3), they slightly decrease the negative charge of the resulting TFN membranes (Figure 8). Also, in the vicinity of positively charged HNTs-G1, the rejection should be governed by the repulsion of positively charged Mg^2+^ cations. In other words, as the HNTs-G1 loading increases, the rejection of MgCl_2_ should increase. This trend is indeed present in Figure 10 despite the size of the error bars.

Considering the high rejection of Na_2_SO_4_ and MgCl_2_, the TFC and TFN membranes fabricated in this study are suitable for water softening, which requires removing multivalent salts. However, due to their unique high rejection of MgCl_2_, a commonly used draw solute, our membranes could also be used in forward-osmosis (FO) separation using MgCl_2_ as a draw solution. High rejection of MgCl_2_ in NF separation promises a low reverse flux of MgCl_2_ in FO applications. Although the rejection of Na_2_SO_4_ by our membranes was higher than MgCl_2_, the former has a higher molecular weight than the latter. Therefore, an aqueous solution of MgCl_2_ of a given weight percentage concentration will create a higher osmotic pressure than an aqueous solution of Na_2_SO_4_ of the same concentration.

It is important to note that most TFN membranes in Table 5 were prepared by adding NPs to the aqueous monomer solution. As the nanoparticles are hydrophilic, it is logical to disperse them in the aqueous monomer solution. However, rubber rolling, which is necessary to remove amine-containing water droplets from the substrate to prevent the formation of micro- or macro-voids in the selective layer, may result in the loss of nanoparticles [49]. Therefore, we dispersed hydrophilic Na_2_SO_4_ in the hydrophobic TMC monomer solution. To minimize the aggregation of hydrophilic NPs in the hydrophobic environment, we used only small loadings of NPs. As a result, although the HNTs-G1 were positively charged (Table 3), the small loadings of NPs did not significantly change the surface zeta potential of the TFN membranes. At the same time, this apparent limitation led to a good balance between the rejection of Na_2_SO_4_ and MgCl_2_. In the case of poly(dopamine) MWCNT, which led to the positive surface zeta potential of the resulting TFN membranes [37], although rejection of MgCl_2_ was the highest in Table 5, rejection of Na_2_SO_4_ dropped below 50%.

## 4. Conclusions

The application of halloysite nanotubes (HNTs) modified with the first generation of poly(amidoamine) (PAMAM) dendrimers as nanoparticles (NPs) was explored for the first time for the fabrication of nanofiltration (NF) thin-film nanocomposite (TFN) membranes. The TFN and reference TFC membranes were synthesized by in situ interfacial polymerization of piperazine (PIP) and 1,3,5-benzenetricarbonyl trichloride (TMC). When synthesizing TFN membranes, the NPs were dispersed in the TMC solution before the polymerization with PIP. The modified HNTs were characterized using ATR-FTIR, TEM, SEM, zeta potential, and thermogravimetric TGA analyses. Fabricated membranes were characterized using SEM, ATR-FTIR, zeta potential, and contact angle measurements. The membranes were also evaluated in cross-flow nanofiltration (NF) tests using 3000 ppm aqueous solutions of MgCl_2_, Na_2_SO_4_ and NaCl, respectively, as feed at 10 bar and ambient temperature.

All membranes showed high rejection of Na_2_SO_4_ (around 97–98%) and low NaCl rejection (less than 40%), with the corresponding water fluxes greater than 100 L m^−2^ h^−1^. The rejection of MgCl_2_ (ranging from 82 to 90%) was less than that of Na_2_SO_4_. However, our values are much greater than those reported in the literature for other TFN membranes. Since, for the same weight percentage in an aqueous solution, osmotic pressure for MgCl_2_ is much greater than for Na_2_SO_4_, the former is more desirable as a draw solute. The remarkable rejection of MgCl_2_ is attributed to the lower negative surface charge of the TFN membranes. Because HNT-G1 nanoparticles are positively charged, their incorporation into the selective layer helps to reject MgCl_2_ at a level acceptable for application as a draw solute. Based on the combination of the water flux and MgCl_2_ rejection, the TFN membrane with 0.05% of nanoparticle loading appears to have the best NF performance.

This work demonstrates the possibility of adjusting the negative surface charge of nanofiltration membranes by using positively charged nanoparticles, which improves the rejection of salts with divalent cations, such as MgCl_2_. The selected nanoparticles, HNTs modified with the first generation of PAMAM dendrimers, are capable of reacting with the organic monomer TMC, allowing their dispersion in the hydrophobic monomer solution despite their hydrophilicity. However, because of the relatively small positive surface charge of HNT-G1 and the small loadings, the changes in the surface charge of the resulting TFN membranes were small. This is a disadvantage, but these small changes in the surface charge of the resulting TFN membranes enabled increasing the rejection of MgCl_2_ without sacrificing the rejection of Na_2_SO_4_.

Future work in this area could include exploring a later generation of PAMAM dendrimers, which could have a greater positive surface charge than HNT-G1 or other positively charged nanoparticles. This would allow more significant changes in the surface charge of the resulting membranes TFN membranes without increasing the loading of nanoparticles. One could also try increasing the loading of positively charged nanoparticles. However, higher loadings of nanoparticles could lead to their aggregation, ultimately resulting in defects in the final TFN membranes that deteriorate their salt-rejection properties. Therefore, any future nanoparticles considered for preparing TFN membranes should have strong interaction (possibly be able to form covalent bonds with one of the monomers) without interfering with the progress of the interfacial polymerization between water-soluble and hexane-soluble monomers.

## Figures and Tables

**Table 1:** Monomer concentration and HNTs-G1 loading for the fabrication of TFC and TFN membranes.

Membrane	PIP in H_2_O (*w*/*v*) %	TMC in n-Hexane (*w*/*v*) %	HNTs-G1 in TMC/n-Hexane (*w*/*v*) %
TFC	2	0.05	0
TFN (0.025%)	2	0.05	0.025
TFN (0.05%)	2	0.05	0.05
TFN (0.1%)	2	0.05	0.1

**Table 2:** Hydrated radius of different salts [31].

Ion	Hydrated Radius (Å)
Cl^−^	3.32
SO_4_^2−^	3.79
Na^+^	3.58
Mg^2+^	4.28

**Table 3:** TGA and Zeta potential results of modified HNTs.

NPs	Weight Loss at 800 °C (%)	Zeta Potential * (mV)
HNTs	17.65	−34.5
HNTs-G1	22.94	+2.2

* Zeta potential determined at neutral pH.

**Table 4:** Total peak area under 1618–1720 cm^−1^ (amide Ι), 1600–1620 cm^−1^ (aromatic amide), and various peaks in the 3120–3706 cm^−1^ range for the PS35 (support), TFC, and TFN membranes.

Membrane	Primary Amide (1618–1720 cm^−1^)	Aromatic Amide (1600–1618 cm^−1^)	3120–3706 cm^−1^
PS35	15.6	2.6	123.2
TFC	16.7	3.2	29.1
TFN (0.025%)	18.4	3.5	36.5
TFN (0.05%)	18.8	3.7	44.3
TFN (0.1%)	17.3	3.3	30.6

**Table 5:** Performance comparison between TFN (HNTs-G1) membrane and other reported TFN membranes.

Nanomaterial	MgCl_2_ Rejection (%)	Na_2_SO_4_ Rejection (%)	Water Permeance (L m^−2^ h^−1^ bar^−1^)	Salt Concentration (g L^−1^)	Ref.
HNTs-G1 *	90.25	96.88	5.65	3	This work
ATP	20	92	23	1	[38]
GO	-	96.56	15.63	1	[39]
TiO_2_ @ GO	6.2	98.8	5.60	1	[40]
GO-COCl *	-	97.1	3.76	1	[41]
ZNGs	41.1	97.8	10.63	1	[42]
COFs (SNW-1)	-	83.5	19.25	1	[43]
SGO	-	96.45	2.37	2.5	[44]
PDA-Si *	68	97	13.33	1	[45]
NH2-SWCNT	51.63	96.34	17.8	2	[46]
Aluminosilicate SWCNT	-	97	<1.2	2	[47]
PMMA- MWNT *	-	99	7	2	[48]
Poly(dopamine) MWCNT	91.5	45.2	15.32	1	[37]

* NPs added into the organic phase; all other NPs were added into the aqueous phase.

## Data Availability

We can make the data available upon request.
